# Enzyme replacement therapy for mucopolysaccharidosis VI: long-term cardiac effects of galsulfase (Naglazyme^®^) therapy

**DOI:** 10.1007/s10545-012-9481-2

**Published:** 2012-06-05

**Authors:** E. Braunlin, H. Rosenfeld, C. Kampmann, J. Johnson, M. Beck, R. Giugliani, N. Guffon, D. Ketteridge, C. M. Sá Miranda, M. Scarpa, I. V. Schwartz, E. Leão Teles, J. E. Wraith, P. Barrios, E. Dias da Silva, G. Kurio, M. Richardson, G. Gildengorin, J. J. Hopwood, M. Imperiale, A. Schatz, C. Decker, P. Harmatz, MPS VI Study Group

**Affiliations:** 1 Pediatric Cardiology, University of Minnesota, Minneapolis, MN USA; 2 Cardiology, Children’s Hospital & Research Center Oakland, Oakland, CA USA; 3 Department of Congenital Heart Diseases / Pediatric Cardiology / GUCH, University Medicine, Center for Diseases in Childhood and Adolescence, Mainz, Germany; 4 Gastroenterology, Children’s Hospital & Research Center Oakland, Oakland, CA USA; 5 Centre for Lysosomal Storage Diseases, University Children’s Hospital, Mainz, Germany; 6 Department of Genetics, UFRGS, Porto Alegre, RS Brazil; 7 Medical Genetics Service, HCPA, Porto Alegre, RS Brazil; 8 INAGEMP – Instituto Nacional de Genética Médica Populacional, Porto Alegre, RS Brazil; 9 Hôpital Femme Mère Enfant, Lyon, France; 10 Metabolic Unit, SA Pathology at Women’s and Children’s Hospital, Adelaide, Australia; 11 Instituto de Biologia Molecular e Celular, Unidade de Biologia do Lisossoma e Peroxisoma, Porto, Portugal; 12 Department of Pediatrics, University of Padova, Padova, Italy; 13 Unidade de Doenças Metabólicas, Departmento de Pediatria, Hospital S. João, Porto, Portugal; 14 Genetic Medicine, St. Mary’s Hospital, Manchester, M13 9WL UK; 15 Cardiologia Pediatrica, Departamento de Pediatria, Hospital de S. João, Porto, Portugal; 16 Cardiology Department, Women’s and Children’s Hospital, Adelaide, Australia; 17 Pediatric Clinical Research Center, Children’s Hospital & Research Center Oakland, Oakland, CA USA; 18 Lysosomal Diseases Research Unit, SA Pathology at Women’s and Children’s Hospital Adelaide, North Adelaide, Australia; 19 BioMarin Pharmaceutical, Inc., Novato, CA USA

## Abstract

Characteristic cardiac valve abnormalities and left ventricular hypertrophy are present in untreated patients with mucopolysaccharidosis type VI (MPS VI). Cardiac ultrasound was performed to investigate these findings in subjects during long-term enzyme replacement therapy (ERT) with recombinant human arylsulfatase B (rhASB, rhN-acetylgalactosamine 4-sulfatase, galsulfase, Naglazyme®). Studies were conducted in 54 subjects before ERT was begun and at specific intervals for up to 96 weeks of weekly infusions of rhASB at 1 mg/kg during phase 1/2, phase 2, and phase 3 trials of rhASB. At baseline, mitral and aortic valve obstruction was present and was significantly greater in those ≥12 years of age. Mild mitral and trace aortic regurgitation were present, the former being significantly greater in those <12 years. Left ventricular hypertrophy, with averaged z-scores ranging from 1.6–1.9 SD greater than normal, was present for ages both <12 and ≥12 years. After 96 weeks of ERT, ventricular septal hypertrophy regressed in those <12 years. For those ≥12 years, septal hypertrophy was unchanged, and aortic regurgitation increased statistically but not physiologically. Obstructive gradients across mitral and aortic valves remained unchanged. The results suggest that long-term ERT is effective in reducing intraventricular septal hypertrophy and preventing progression of cardiac valve abnormalities when administered to those <12 years of age.

## Introduction

Mucopolysaccharidosis type VI (MPS VI, Maroteaux-Lamy syndrome) is a lysosomal storage disease caused by functional absence of the enzyme N-acetylgalactosamine 4-sulfatase (arylsulfatase B or ASB; E.C. 3.1.6.12). Absence of this enzyme results in the accumulation of dermatan-sulfated glycosaminoglycans (GAGs) within lysosomes of various tissues including bones, cartilage, lungs, airways, and the cardiovascular system (Neufeld and Muenzer 2001). The progressive accumulation of these substances results in multi-organ system dysfunction such as joint contractures, short stature, dysostosis multiplex, decreased pulmonary function, cardiac abnormalities and, ultimately, shortened life span (Neufeld and Muenzer 2001). The severity of the clinical findings is variable but, in a recent survey of 121 MPS VI subjects, an accelerated clinical course was associated with urinary excretion of GAGs in excess of 200 μg/mg creatinine (Swiedler et al. 2005).

Evaluation of the heart in individuals with untreated MPS VI by cardiac ultrasound has been reported by several investigators (Lael et al. 2010; Dangel 1998; Wippermann et al. 1995; Azevedo et al. 2004; Scarpa et al. 2009; Fesslova et al. 2009). The characteristic cardiac abnormalities of MPS VI include ventricular hypertrophy and a progressive thickening of mitral and aortic valves resulting in valvular regurgitation, stenosis, or both. Until now the effects of enzyme replacement therapy (ERT) upon the heart in MPS VI have not been reported.

Previous phase 1, 2, and 3 studies have shown that treatment of individuals with MPS VI by ERT with recombinant human N-acetylgalactosamine 4-sulfatase (rhASB; galsulfase; Naglazyme®) is safe, rapidly reduces urinary GAG levels, and improves endurance as measured by 6- or 12-min walk and pulmonary function testing (Harmatz et al. 2004, 2005, 2006). Further analysis of pooled data from the clinical ERT trials and the survey study has demonstrated safety and significant long-term increases in endurance, pulmonary function, and growth when galsulfase is administered for 96 weeks or more (Harmatz et al. 2008, 2010; Decker et al. 2010). The purpose of this report is to analyze cardiac ultrasound data obtained during phase 1, 2, and 3 clinical ERT trials to determine the effects of 96 weeks of ERT upon the characteristic cardiac findings in individuals with MPS VI.

## Methods

Previous reports have detailed the study design and outcomes of the phase I/2, 2, and 3 galsulfase trials in subjects with MPS VI (Harmatz et al. 2004, 2005, 2006, 2008, 2010); details of the clinical trials are outlined in Table 1. Cardiac ultrasound was performed as part of the clinical evaluation of subjects in the phase 1/2, 2, and 3 clinical trials at baseline, before ERT was begun, and at intervals of 24–48 weeks and 72–96 weeks after initiation of ERT (Table 2). An Institutional Review Board (IRB) or Ethics Committee (EC) at each participating clinical site approved each study. All adult patients and parent/guardians gave written consent; patients younger than 18 years old gave written assent according to local IRB regulations.

**Table 1:** Summary of study populations

Study	Study design	Study dates	Study duration (weeks)	Patients enrolled/completed	Dose of rhASB (mg/kg)	Age (years), mean ± SD (range)	Sex (M/F)
Phase 1/2	Double-blinded, randomized, dose comparison/open-label extension	Jan 2001–Dec 2005	240	7/5	0.2	12.0 ± 3.8 (7–16)	4/3
Phase 2	Open-label, nonrandomized	2002–2006	144	10/10	1.0	12.1 ± 5.3 (6–21)	7/3
Phase 3	Double-blinded, placebo-controlled, randomized/open-label extension	2003–2006	96	39/38	1.0	13.7 ± 6.5 (rhASB); 10.7 ± 4.4 (placebo); (6–29)	13/26

*rhASB* Recombinant human arylsulfatase B

**Table 2:** Schedule of cardiac ultrasound evaluations

Study	Treatment week				
	0	24	48	72	96
Phase 1/2	X	X	X	X	X
Phase 2	X		X		X
Phase 3 rhASB/rhASB	X		X		X
Phase 3 placebo/rhASB	X	X		X	

*rhASB* Recombinant human arylsulfatase B

Archived ultrasound data, collected from subjects who had participated in phase 1/2, 2, or 3 clinical trials, were reviewed and tabulated for this study. The original echoes were obtained and analyzed at the individual sites; original echo tapes were unavailable for further review. Each archived measurement was reviewed for reliability by a single person (E.B.). Discrepancies were resolved by discussion with individual sites. The entire data set was then subjected to statistical evaluation. Height, weight, and urinary GAG content at study entry were analyzed for the 54 subjects who participated in phase 1/2, 2, or 3 galsulfase trials.

Cardiac ultrasound investigations were performed and interpreted by study protocol at the participating sites during each study point as indicated in Table 2. Measurements included M-mode determination of left ventricular chamber dimension in diastole (LVED) and systole (LVES), as well as diastolic left ventricular posterior wall (LVPWd) and intraventricular septal (IVSd) thicknesses. Body surface areas were calculated by the method of duBois for each subject, and z-scores were determined from the measured value for each chamber dimension and wall thickness at each evaluation (Dyar 2012). The individual z-scores from each subject for each parameter at each time point were summed and averaged to obtain the mean z-score displayed in the tables. By use of the z-score, chamber dimensions and wall thicknesses from subjects of differing body surface areas can be compared, and the changes within a particular subject tracked over time (Kampmann et al. 2000). With this system, a z-score of 0 represents the expected normal value for a given body surface area, a positive z-score is a standard deviation value greater than expected normal, and a negative z-score is a value less than expected normal. Left ventricular shortening fraction (SF) was calculated by standard methods (Lopez et al. 2010).

Doppler interrogation of flow acceleration across, and regurgitation from, all cardiac valves was measured by pulsed, continuous, and color flow Doppler methods. Peak systolic gradient was recorded across aortic valves; mean diastolic gradient was measured across mitral valves; and both were compared to normal values (Lopez et al. 2010; Hatle and Angelsen 1985; Baumgartner et al. 2009; Sohn and Kim 2001). Valvular regurgitation was assigned the following scores: 0 (none), 1 (trace), 2 (mild), 3 (moderate), and 4 (severe). Mitral and aortic valve gradients and regurgitation scores for the cohort were recorded at baseline, 48 and 96 weeks. Three subjects had undergone mitral valve repair or replacement (two before and one during the trials) or aortic valvuloplasty (one before the trials). For purposes of this review, peak systolic aortic gradient was the only variable analyzed after mitral valve replacement in these three subjects. Additional cardiac anomalies were noted when found.

Ultrasound reports were obtained and reviewed retrospectively. Data from the phase I/2 study and phase 3 study were analyzed for differences between combined reduced dose (0.2 mg/kg, phase 1/2 low-dose group) or delayed dose (phase 3, placebo group) and standard dose (1 mg/kg) treatment. Data were pooled from all three treatment trials (phase 1/2, phase 2, and phase 3) and then analyzed for baseline values and for differences between baseline and 24–48 and 72–96 weeks of treatment with galsulfase.

Data were analyzed using all available data and then re-analyzed using only those subjects for whom data were available at all three study points (baseline, weeks 48 and 96) of galsulfase treatment.

### Statistical analysis

Initial analyses were based on analysis of variance models or Student’s *t*-test to assess differences in demographic variables at baseline between the various subgroups compared. Analysis of differences by gender and by age (<12 years versus ≥12 years) were made both at baseline and between weeks 24–48 and 72–96. Finally, the major hypotheses were evaluated using general linear models and performed for all subjects for whom data were available and again for subjects for whom data were available at all three study points of galsulfase treatment (Pettersen et al. 2008; Diggle et al. 2012). The models investigated differences over the three time points between and within each of the two treatment groups (reduced or delayed vs. standard dose). To estimate more efficient and unbiased regression parameters of data collected as repeated measures over time, the generalized estimating equation approach of Zeger and Liang was used (Diggle et al. 2012). This allowed specification of a working correlation matrix that accounts for the within-subject correlations. A significance level of 0.05 was used for all statistical tests. Data were analyzed using SAS version 9.2.

## Results

### Baseline studies

The mean age for the entire group was 11.8 ± 5.4 (range 6–29) years; 37 (68 %) were females and 17 (32 %) males. The mean urinary GAG of 341.4 ± 120.5 μg GAG/mg creatinine was elevated and concomitant mean age-adjusted stature (102.5 ± 13 cm) reduced (Table 3), suggesting more severe MPS VI disease in this group of individuals as has been previously reported by others (Swiedler et al. 2005). Height, weight, and body surface area differed by age (*p* = 0.008, 0.0008, and 0.001, respectively, data not shown); however, there was no difference in height, weight, or urinary GAG either by gender (*p* = 0.373, 0.373, and 0.235, respectively) or in GAG by age (*p* = 0.224).

**Table 3:** Demographics

Subjects	Baseline height (cm)	Baseline GAG (μg glycosaminoglycan/mg creatinine)
	Mean ± SD	Mean ± SD
All (*n* = 54)	102.54 ± 12.99	341.43 ± 120.53
<12 years (*n* = 33)	98.9 ± 11.0**	357.5 ± 119.5
≥12 years (*n* = 21)	108.3 ± 13.98**	316.23 ± 120.65
Male (*n* = 17)	104.9 ± 13.4	312.5 ± 101.4
Female (*n* = 37)	101.5 ± 12.8	354.72 ± 127.44

**p = 0.008 for height by age groups

#### Cardiac dimensions and function

Cardiac findings for the 54 subjects before the initiation of ERT are listed in Table 4. Males and females did not significantly differ in age (12.2 ± 6 vs. 11.7 ± 5.2 years, *p* = 0.742) or in any measured cardiac parameter (data not shown), thus both genders were grouped together for all subsequent analyses. The mean z-score of left ventricular end-diastolic dimension (LVED) was within 1 SD of normal at baseline for all participants, regardless of age. By contrast, the mean z-score of LVES was 1.2 SD smaller than normal in those ≥12 years of age, a finding that just reached statistical significance (*p* = 0.039) when compared to those <12 years. Despite this finding, SF was within normal limits for all participants, regardless of age. The mean z-scores for left ventricular wall thicknesses (LVPWd, IVSd) were increased to 1.4 –1.9 SD greater than normal at baseline for all participants regardless of age (*p* = 0.858 and 0.229, respectively) in keeping with the infiltrative nature of the lysosomal storage diseases.

**Table 4:** Baseline data from 54 MPS VI subjects enrolled in galsulfase trials

Measurement	Normal value	Total	<12 years	≥12 years	*p* value
		*n* = 54	*n* = 33	*n* = 21	
Age	-	11.8 ± 5.4 (*n* = 54)	8.3 ± 1.6 (*n* = 33)	17.3 ± 4.6 (*n* = 21)	
LVED mean z-score	0	−0.16 ± 1.25 (*n* = 54)	0.08 ± 1.33 (*n* = 33)	−0.54 ± 1.02 (*n* = 21)	0.076
LVES mean z-score	0	−0.74 ± 1.40 (*n* = 54)	−0.43 ± 1.31 (*n* = 33)	−1.23 ± 1.43 (*n* = 21)	0.039*
SF (%)	≥28	42.20 ± 6.63 (*n* = 54)	41.49 ± 6.28 (*n* = 33)	43.31 ± 7.15 (*n* = 21)	0.331
LVPWd mean z-score	0	1.86 ± 0.99 (*n* = 53)	1.88 ± 1.08 (*n* = 33)	1.83 ± 0.85 (*n* = 20)	0.858
IVSd mean z-score	0	1.57 ± 0.95 (*n* = 54)	1.69 ± 0.99 (*n* = 33)	1.37 ± 0.88 (*n* = 21)	0.229
AoPSG (mmHg)	<10	13.72 ± 10.78 (*n* = 34)	9.24 ± 3.90 (*n* = 18)	18.77 ± 13.68 (*n* = 16)	0.015*
MMV (mmHg)	<5	7.28 ± 5.68 (*n* = 32)	5.15 ± 3.98 (*n* = 17)	9.69 ± 6.46 (*n* = 15)	0.022*
Aortic regurgitation	0	0.53 ± 0.87 (*n* = 54)	0.68 ± 0.97 (*n* = 33)	0.29 ± 0.64 (*n* = 21)	0.104
Mitral regurgitation	0	1.35 ± 1.01 (*n* = 51)	1.58 ± 1.01 (*n* = 31)	1.00 ± 0.93 (*n* = 20)	0.044*

*LVED* Left ventricular chamber dimension in diastole, *LVES* left ventricular chamber dimension in systole, *SF* shortening fraction, *LVPWd* diastolic left ventricular posterior wall thickness, *IVSd* diastolic intraventricular septal thickness, *AoPSG* aortic peak systolic gradient, *MMV* mean mitral valve gradient

*p-value comparing age <12 years to ≥12 years

#### Cardiac valves

Before ERT, the mean mitral valve gradient of 7.28 ± 5.68 mmHg was higher than normal (<5 mmHg) (Lopez et al. 2010; Hatle and Angelsen 1985; Baumgartner et al. 2009; Sohn and Kim 2001), indicating obstruction to flow (mitral stenosis) prior to institution of galsulfase therapy. The mitral gradient was significantly greater for subjects ≥12 vs. <12 years (9.69 ± 6.46 vs. 5.15 ± 3.98 mmHg, respectively, *p* = 0.022) (Table 4). Two of the three mitral valve replacements were performed in those >12 years of age. For the entire cohort, the mitral valve was mildly regurgitant at baseline (score 1.35 ± 1.05). In contrast to mitral stenosis, mitral regurgitation was significantly less in older subjects (mitral regurgitation score 1.00 ± 0.93 vs. 1.58 ± 1.01 for subjects ≥12 vs. <12 years, *p* = 0.044).

The aortic valve was less affected than the mitral before ERT. The peak systolic gradient across the aortic valve for the entire group of 13.72 ± 10.78 mmHg was at the upper limit of childhood normal values (11–13 mmHg) (Lopez et al. 2010; Hatle and Angelsen 1985; Baumgartner et al. 2009; Sohn and Kim 2001). As with the mitral valve, the aortic gradient was significantly greater in older participants (18.77 ± 13.68 vs. 9.24 ± 3.90 mmHg, *p* = 0.015). Aortic valvuloplasty was performed in one subject >12 years of age before the initiation of therapy. Aortic regurgitation was barely perceptible at baseline (aortic regurgitation score of 0.53 ± 0.87 equivalent to trace regurgitation) and did not differ between older and younger subjects (*p* = 0.104 for age ≥12 vs. <12 years).

#### Other cardiac anomalies

Other cardiac anomalies were rare in this group of subjects, but included one subject with partial anomalous pulmonary venous return, sinus venosus atrial septal defect, and pulmonary stenosis, who had undergone pulmonary valvotomy.

### Studies after galsulfase treatment

#### Cardiac dimensions and function

Data from the entire cohort are presented in Table 5, while data from subjects in whom all three time points were available (before ERT, weeks 24–48, and weeks 72–96) are presented in Table 6. After 96 weeks of enzyme, the mean z-score for LVED remained unchanged (*p* = 0.150, *p* = 0.146, respectively) and normal (Table 5, Table 6) for both groups. The mean z-score for left ventricular systolic dimension (LVES) increased significantly (*p* = 0.034) during therapy for the entire cohort but not for those in whom all three time points were available (*p* = 0.053), but this change did not adversely affect cardiac function since the SF remained normal for both groups throughout the study (*p* = 0.208, *p* = 0.135, respectively). For both the entire cohort and for those in whom all three time points were available, the mean z-score for left ventricular posterior wall thickness (LVPWd) remained increased and did not change (*p* = 0.551, *p* = 0.510, respectively) after 96 weeks of enzyme, but the mean z-score for IVSd decreased significantly (*p* < 0.0001and *p* < 0.0001, respectively) during this period. One subject was lost to follow-up after baseline studies. Analysis of data with and without his inclusion did not alter results. Prior to combining data from the two groups, we had examined all data as a function of time, comparing standard vs. reduced or delayed enzyme. We observed a significant time versus group interaction (*p* = 0.0396) for IVSd (Table 5, footnote 1, Figure A).

**Table 5:** ECHO data from MPS VI subjects during galsulfase trials

Echo parameter	Baseline (*n* = 54)	24–48 weeks (*n* = 53)	72–96 weeks (*n* = 52)	*p*-value (based on repeated measures model comparing all three time points)
LVED mean z-score	−0.16 ± 1.25 (*n* = 54)	0.11 ± 1.17 (*n* = 53)	−0.10 ± 1.15 (*n* = 51)	0.150
LVES mean z-score	−0.74 ± 1.40 (*n* = 54)	−0.28 ± 1.37* (*n* = 52)	−0.53 ± 1.30 (*n* = 51)	0.034
SF (%)	42.20 ± 6.63 (*n* = 54)	40.35 ± 6.65 (*n* = 53)	40.40 ± 8.11 (*n* = 51)	0.208
LVPWd mean z-score	1.86 ± 0.99 (*n* = 53)	1.62 ± 1.94 (*n* = 53)	1.64 ± 0.97 (*n* = 51)	0.551
IVSd mean z-score	1.57 ± 0.95 (*n* = 54)	1.14 ± 0.86* (*n* = 53)	0.97 ± 0.89* (*n* = 51)	<0.0001^a^
AoPSG (mmHg)	13.72 ± 10.78 (*n* = 34)	11.78 ± 6.89 (*n* = 37)	12.99 ± 9.00 (*n* = 34)	0.150
MMV (mmHg)	7.28 ± 5.68 (*n* = 32)	7.75 ± 6.63 (*n* = 34)	7.13 ± 5.64 (*n* = 30)	0.552
Aortic regurgitation	0.53 ± 0.87 (*n* = 54)	0.73 ± 0.97* (*n* = 53)	0.88 ± 0.99* (*n* = 50)	0.004^b^
Mitral regurgitation	1.35 ± 1.01 (*n* = 51)	1.42 ± 0.89 (*n* = 51)	1.51 ± 0.73 (*n* = 47)	0.459

*LVED* Left ventricular chamber dimension in diastole, *LVES* left ventricular chamber dimension in systole, *SF* shortening fraction, *LVPWd* diastolic left ventricular posterior wall thickness, *IVSd* diastolic intraventricular septal thickness, *AoPSG* aortic peak systolic gradient, *MMV* mean mitral valve gradient

*p < 0.05 compared to baseline

^a^We observed a significant time versus group interaction (*p* = 0.0396) for IVSd. Data shown in Figure A as mean ± SD

^b^We observed a significant time versus group interaction (*p* = 0.019) for aortic regurgitation. Data shown in Figure B as mean ± SD

**Table 6:** ECHO data from MPS VI subjects during galsulfase trials in which subjects had data on all three time points for each particular variable

ECHO	Baseline (mean ± SD)	48 weeks (mean ± SD)	96 weeks (mean ± SD)	ANOVA *p*-value comparing weeks 0, 48, 96
LVED mean z-score (*n* = 50)	−0.13 ± 1.20	0.14 ± 1.10	−0.11 ± 1.16	0.146
LVES mean z-score (*n* = 49)	−0.76 ± 1.39	−0.31 ± 1.37	−0.55 ± 1.31	0.053
SF % (*n* = 50)	42.58 ± 6.71	50.73 ± 6.54	39.72 ± 9.95	0.135
LVPWd mean z-score (*n* = 49)	1.91 ± 1.00	1.63 ± 2.00	1.68 ± 0.96	0.510
IVSd mean z-score (*n* = 50)	1.61 ± 0.97	1.13 ± 0.86	0.99 ± 0.88	<0.0001
AoPSG (mmHg) (*n* = 27)	13.28 ± 11.92	11.81 ± 7.48	12.49 ± 10.20	0.516
MMV (mmHg) (*n* = 28)	7.19 ± 4.61	7.14 ± 5.31	7.26 ± 5.77	0.971
Aortic regurgitation (*n* = 48)	0.53+ 0.83	0.70 ± 0.93	0.88 ± 0.99	0.008
Mitral regurgitation (*n* = 43)	1.33 ± 0.96	1.34 ± 0.85	1.48 ± 0.75	0.402

*LVED* Left ventricular chamber dimension in diastole, *LVES* left ventricular chamber dimension in systole, *SF* shortening fraction, *LVPWd* diastolic left ventricular posterior wall thickness, *IVSd* diastolic intraventricular septal thickness, *AoPSG* aortic peak systolic gradient, *MMV* mean mitral valve gradient

**Table 7:** ECHO data from MPS VI subjects by age during galsulfase trials

Echo parameter	Age <12 years		*p*-value	Age ≥12 years		*p*-value
	Baseline	96 weeks		Baseline	96 weeks	
LVED mean z-score	0.08 ± 1.33 (*n* = 33)	−0.30 ± 1.15 (*n* = 25)	0.219	−0.54 ± 1.02 (*n* = 21)	−0.09 ± 1.14 (*n* = 26)	0.584
LVES mean z-score	−0.43 ± 1.31 (*n* = 33)	−0.59 ± 1.19	0.830	1.37 ± 0.88 (*n* = 21)	1.04 ± 0.91 (*n* = 26)	0.265
SF (%)	41.49 ± 6.28 (*n* = 33)	38.74 ± 9.80	0.247	43.31 ± 7.15 (*n* = 21)	42.00 ± 5.83 (*n* = 26)	0.747
LVPWd mean z-score	1.88 ± 1.08 (*n* = 33)	1.56 ± 1.03 (*n* = 25)	0.368	1.83 ± 0.85 (*n* = 20)	1.72 ± 0.93 (*n* = 26)	0.980
IVSd mean z-score	1.69 ± 0.99 (*n* = 33)	0.89 ± 0.87 (*n* = 25)	<0.0001	1.37 ± 0.88 (*n* = 21)	1.04 ± 0.91 (*n* = 26)	0.318
AoPSG (mmHg)	9.24 ± 3.90 (*n* = 18)	10.06 ± 4.48 (*n* = 18)	0.993	18.77 ± 13.68 (*n* = 16)	16.29 ± 12.33 (*n* = 16)	0.790
MMV (mmHg)	5.15 ± 3.98 (*n *= 17)	6.57 ± 6.80 (*n* = 14)	0.3745	9.69 ± 6.46 (*n* = 15)	7.62 ± 4.58 (*n* = 16)	0.999
Aortic regurgitation	0.68 ± 0.97 (*n* = 33)	0.81 ± 0.83	0.080	0.29 ± 0.64 (*n* = 21)	0.94 ± 1.13 (*n* = 26)	0.015
Mitral regurgitation	1.58 ± 1.01 (*n* = 31)	1.56 ± 0.68 (*n* = 24)	0.983	1.00 ± 0.93 (*n* = 20)	1.46 ± 0.78 (*n* = 23)	0.218

*LVED* Left ventricular chamber dimension in diastole, *LVES* left ventricular chamber dimension in systole, *SF* shortening fraction, *LVPWd* diastolic left ventricular posterior wall thickness, *IVSd* diastolic intraventricular septal thickness, *AoPSG* aortic peak systolic gradient, *MMV* mean mitral valve gradient

Because there was no statistical difference in wall thickness between the entire cohort and those in whom all three measurement points were available, we analyzed the data for those <12 years of age vs. those ≥12 years of age from the entire cohort. Treatment with galsulfase appeared to have more effect when administered to those <12 years of age (Table 7). For those subjects <12 years of age, the mean z-score for IVSd reflected significantly less hypertrophy (*p* < 0.0001) while for those ≥12 years it did not change (*p* = 0.318).

#### Cardiac valves

After 96 weeks of enzyme, neither mitral valve obstruction (MMV) nor mitral valve regurgitation (MRS) changed in either the entire cohort or in those in whom all three measurement points were available (MMV: *p* = 0.552, *p* = 0.971, respectively, and MRS: *p* = 0.459, *p* = 0.402, respectively, Tables 5 and 6). Aortic valve obstruction remained unchanged for both the entire cohort and for those in whom all three time points were available (*p* = 0.150, *p* = 0.516, respectively) during the studies, but aortic regurgitation increased significantly (*p* = 0.004, *p* = −0.008, respectively) while still remaining within the “trace to mild” category. As discussed above, prior to combining data from the two groups, we examined the pattern over time for each group (standard vs. reduced or delayed) to learn if it was similar for the two groups. We observed a significant time versus group interaction (*p* = 0.019) for aortic regurgitation (Table 5, footnote 2, Figure B) although again we could not identify any reasonable explanation as to why this would be the case.

When the entire cohort was analyzed by age <12 vs. ≥12 years, neither mitral nor aortic valve stenosis changed after 96 weeks of therapy. Mitral regurgitation did not increase in either age group (Table 7) after 96 weeks of treatment, but aortic valve regurgitation score increased in those ≥12 years of age (*p* = 0.015).

## Discussion

The cardiovascular system is progressively and unambiguously affected in individuals with MPS VI. Left ventricular hypertrophy, as well as anatomic and functional abnormalities of the mitral and aortic valves, have previously been well described by others (Lael et al. 2010; Dangel 1998; Wippermann et al. 1995; Azevedo et al. 2004; Scarpa et al. 2009; Fesslova et al. 2009). Our data obtained prior to the initiation of ERT from a large number of severely affected subjects with MPS VI support these observations but, more importantly, describe the cardiac effects of long-term treatment with galsulfase ERT.

Prior to ERT, left ventricular hypertrophy and mitral valve stenosis were the most prominent cardiac features found by this study. Left ventricular hypertrophy was severe, with mean z-scores approaching 2 SD greater than normal, and was found in subjects of all ages. The mean gradient across the mitral valve was elevated at baseline and increased significantly when those <12 years of age were compared to those ≥12. Mitral valve replacement, performed in three subjects before, or during, these studies confirmed the severity of this finding. By contrast, mitral valve regurgitation, usually a more common finding in most MPS VI pediatric reports (Lael et al. 2010; Dangel 1998; Wippermann et al. 1995; Azevedo et al. 2004; Scarpa et al. 2009; Fesslova et al. 2009) was only mild in our subjects. The presence of mitral stenosis, rather than regurgitation, has been found in older individuals with MPS VI (Diggle et al. 2012; Marwick et al. 1992; Tan et al. 1992) and is consistent with the older age of the subjects in this study.

Aortic valve obstruction at baseline was significantly greater in those 12 years of age or older but, when compared to mitral obstruction, was milder. Only one subject underwent aortic valvuloplasty prior to initiation of enzyme treatment. Only trace aortic regurgitation was present at baseline, a finding considered not physiologically significant.

Long-term enzyme replacement therapy with galsulfase was associated with maintenance of normal left ventricular function in all subjects in this study and regression of left ventricular septal hypertrophy in those who initiated treatment before 12 years of age. This remained true when we evaluated the entire cohort as well as when we analyzed only those in whom all three measurement points were available. During the course of this study, cardiac valve stenosis neither worsened nor improved, regardless of age. Aortic valve regurgitation increased significantly—but not physiologically—after 96 weeks of enzyme replacement in subjects ≥12 years of age. Although this finding had little physiologic consequence to these individuals, it may imply that cardiac valve pathology, once begun, may not be reversible. This progression of aortic regurgitation in the ≥12 years of age group is most likely due to underlying disease. It is difficult to assess causal relationship with ERT treatment given that the treatment group was followed for 96 weeks, placebo for 24 weeks, and echocardiography was not assessed after 24 weeks of treatment or placebo.

Valve obstruction is identified by the measurement of increased Doppler flow velocities across cardiac valves. With a normal cardiac output, the maximum normal Doppler velocity across the mitral valve is 1.3 m/s or 6.7 mmHg in children (Hatle and Angelsen 1985). The mean mitral gradient, a more accurate measure of obstruction, is obtained by averaging the instantaneous mitral flow velocities throughout diastole, resulting in a lower value. Mean mitral valve gradients <5 mmHg are consistent with mild mitral obstruction in adults; no values have been established for children (Baumgartner et al. 2009). The maximum normal Doppler velocities across the aortic valve in adults and children are 1.7–1.8 m/s, respectively, corresponding to peak aortic gradients of 11–13 mmHg (Hatle and Angelsen 1985). At baseline the mean mitral and aortic valve gradients in our subjects exceeded normal values and were greater in older subjects, consistent with the progressive nature of MPS VI.

The lack of response of the cardiac valves to ERT is similar to that reported in a small series of MPS VI patients who underwent hematopoietic stem cell transplantation (HSCT) and were studied an average of 5 (range 1.8–9) years after the procedure (Herskhovitz et al. 1999). The relatively avascular nature of cardiac valve tissue (Dow and Harper 1932) may, in part, explain the lack of improvement in valve morphology and function with either HSCT or ERT. Irreversible valve damage accruing over years may also explain the lack of response in older children and young adults (such as the subjects of this study) to any type of intervention. In support of early intervention, galsulfase, given from 8 weeks of life, has been shown to prevent cardiac abnormalities altogether (McGill et al. 2010).

Although this study had a large number of subjects with MPS VI who were studied over a lengthy time period, there are limitations to the study. The placebo group was followed for only the first 24 weeks of the phase 3 study. If follow-up had been extended for a longer time period, other differences between the placebo and treated groups may have been identified. The second limitation of this study is that ultrasounds were performed and analyzed at local sites. The concept of a central echocardiographic facility to provide reliable and reproducible data for multicenter pediatric cardiac studies emerged during the course of these trials (Lipschultz et al. 2001). Cardiac ultrasound in subjects with MPS can be difficult due to abnormalities of the thorax, poor lung expansion from hepatomegaly and restrictive lung disease, and inability to extend the neck. Comparison of M-mode measurements made in the field versus the central location in two different pediatric studies (Lipschultz et al. 2001; Dai et al. 1999) suggests that repeated measurements from the field may be more reliable than a single measurement. Thus although inter-institutional differences may have affected the absolute values obtained in this study, it is likely that trends (repeated values from the same sites) were less affected.

## Summary

Left ventricular hypertrophy and significant mitral valve obstruction are reported in 54 individuals with MPS VI ranging from 6 to 29 years of age undergoing treatment trials with galsulfase. Long-term enzyme replacement with galsulfase was associated with stable left ventricular function in all subjects and regression of intraventricular septal hypertrophy when enzyme therapy began before the age of 12 years. Despite long-term enzyme therapy, cardiac valve stenosis remained unchanged in all subjects. Aortic regurgitation increased statistically, but not physiologically, only in those who began enzyme at 12 or more years of age. The evidence presented suggests that the underlying pathological changes due to GAG accumulation, especially in the valves, may be poorly reversible and that early initiation of galsulfase therapy may be beneficial.
